# Calcium Channel Blocker Overdose Causes Acute Respiratory Distress Syndrome and Acute Kidney Injury in a 15-Year-Old Female

**DOI:** 10.7759/cureus.43806

**Published:** 2023-08-20

**Authors:** Larsen H Welsh, Jeremy T Bose, Hanna S Sahhar

**Affiliations:** 1 Pediatrics, Edward Via College of Osteopathic Medicine, Spartanburg, USA; 2 Pediatric Intensive Care Unit, Spartanburg Regional Healthcare System, Spartanburg, USA

**Keywords:** amlodipine toxicity, pediatrics, calcium channel blocker overdose, acute respiratory distress syndrome, acute kidney injury

## Abstract

Calcium channel blockers (CCBs) are among the most commonly prescribed cardiovascular medications in the adult population. Approximately 20% of adults with hypertension in the United States are prescribed dihydropyridine calcium channel blockers. Similarly, in the pediatric population, CCBs such as nifedipine and amlodipine are frequently prescribed in the non-emergent management of hypertension in children and adolescents. Despite the prevalence of CCB usage, the available literature on the management of calcium channel blocker toxicity in the pediatric population remains scarce. In the absence of formal guidelines, the management of CCB overdoses comes from case reports.

This case identifies a 15-year-old Hispanic female who developed acute respiratory distress syndrome (ARDS) and acute kidney injury (AKI) after an overdose of amlodipine. Our patient presented with profound, refractory hypotension requiring substantial inotropic support. She subsequently developed significant dyspnea, desaturating into the 80s with radiological evidence of ARDS requiring endotracheal intubation. After aggressive diuresis and electrolyte replacement, along with inotropic agents to maintain adequate blood pressure, our patient began to make significant clinical progress. With continued improvement and resolution of her AKI and ARDS, she was successfully weaned off ventilatory support and all infusions. Our patient was deemed medically appropriate for discharge 10 days after the initial presentation and was admitted to an inpatient psychiatric unit.

Calcium channel blocker toxicity can pose considerable risks, as was seen with our patient. Prompt recognition and judicious management of CCB overdoses can mitigate associated morbidity and mortality, resulting in favorable outcomes for patients. The intention behind documenting this case is to contribute to the limited literature on the successful management of calcium channel blocker poisoning in the pediatric population.

## Introduction

As the prevalence of cardiovascular disease increases, calcium channel blockers (CCBs) remain among the most commonly prescribed cardiovascular medications in the adult population. In the pediatric population, CCBs such as nifedipine and amlodipine are popularly prescribed in managing pediatric hypertension. Fortunately, calcium channel blockers are generally well tolerated when ingested as intended. Common adverse effects include headaches, flushing, peripheral edema, and gastrointestinal upset [1]. Intentional or inadvertent overdoses of CCBs can lead to severe toxicity and life-threatening complications such as hypotension, dysrhythmias, and hyperglycemia [2].

Remarkably, no formal guidelines outlining the management of CCB poisoning currently exist, despite the significant morbidity and mortality associated with calcium channel blocker toxicity [3]. The available literature on treatment strategies for pediatric patients remains especially scarce. The management of calcium channel blocker toxicity in this population is primarily limited to case reports and expert consensus recommendations for CCB poisoning in adults [2]. The presentation of calcium channel blocker toxicity can vary greatly depending on the age of the patient, the quantity ingested, and co-ingestions, as is the case with our patient. Presenting symptoms can range from dizziness and fatigue to altered mental status and shock [4]. Dihydropyridine calcium channel blockers such as amlodipine can cause profound hypotension through peripheral arterial vasodilation, reducing peripheral vascular resistance. Severe and refractory hypotension can eventually lead to end-organ dysfunction such as acute kidney injury (AKI) and acute respiratory distress syndrome (ARDS) [5]. Inadequate tissue perfusion can also lead to metabolic acidosis secondary to lactic acidosis [6]. Metabolic abnormalities such as hyperglycemia may occur as calcium channel blockers reduce pancreatic insulin release and increase insulin resistance [6].

The initial treatment for critical CCB toxicity includes airway maintenance and circulatory support. Severe hypotension can be managed with intravenous fluids, calcium, and inotropes such as dopamine, norepinephrine, and epinephrine [7]. Gastrointestinal decontamination with activated charcoal should also be considered in patients presenting within one hour of ingestion [2]. Metabolic abnormalities and acid-base disturbances must also be corrected in the management of CCB overdose with agents such as glucagon, high-dose insulin, and lipid emulsion therapy [3].

## Case presentation

A 15-year-old Hispanic female with no significant medical history presented to the emergency department (ED) after ingesting 20 tablets of 10 mg amlodipine one day prior and 14 tablets of 5 mg tizanidine two days prior to admission. The morning after ingesting 200 mg of amlodipine, she began to experience mild chest pain, nausea, multiple episodes of vomiting, and dizziness, which prompted her visit to the ED.

Upon initial examination in the ED, the patient was not in acute distress. Her blood pressure was 91 mmHg systolic over 38 mmHg diastolic; her heart rate was 120 beats per minute; she had a temperature of 97.9°F and a body weight of 63.7 kg. Notably, the patient's distal pulses were 2+ and symmetric. Laboratory analysis revealed an elevated white blood cell count of 18.8 x 103/uL as well as a hemoglobin of 10.8 g/dL. The patient was also found to have a creatinine of 2.04 mg/dL, a blood sugar level of 182 mg/dL, and a calcium level of 9.7 mg/dL. An electrocardiogram showed no abnormalities.

The patient received 3 liters of intravenous normal saline and 1 gram of calcium gluconate. Unfortunately, she remained profoundly hypotensive and tachycardic and was subsequently admitted to the pediatric intensive care unit (PICU) for inotropic support. Upon admission to the PICU, a 0.3 mcg/kg/min norepinephrine drip was started. Glucagon was held at this time due to the patient’s severe emesis.

Over the next 24 hours, the patient developed significant dyspnea. Subsequent radiologic findings revealed bilateral pleural effusions most suggestive of pulmonary edema, with the possibility of aspiration in the setting of persistent emesis. The patient also became oliguric and experienced a considerable increase in her creatinine, peaking at 3.33 mg/dL. This acute kidney injury (AKI) was likely secondary to severe hypotension due to the vasodilatory effects of CCBs. The patient’s norepinephrine drip was increased to 1.6 mcg/kg/min, and she was transferred to a higher-level facility due to the possible need for renal dialysis.

Upon arrival, the patient was in significant respiratory distress with desaturations in the 80s. She required emergent sedation and endotracheal intubation. A central venous line and a nasogastric tube were also placed. The patient was on continuous mandatory ventilation (CMV) with the following settings: tidal volume of 450 mL, positive end-expiratory pressure (PEEP) of 12, fraction of inspired oxygen (FiO_2_) of 70% with oxygen saturation (SpO_2_) in the low 90s, and a set respiratory rate of 12 br/min. Venous blood gas analysis revealed a potential of hydrogen (pH) of 7.30, a partial pressure of carbon dioxide (PCO_2_) of 43 mmHg, a partial pressure of oxygen (pO_2_) of 50 mmHg, and bicarbonate (HCO3) of 21.0 mmol/L.

Laboratory investigations revealed persistently elevated lactate, peaking at 7.4 (normal reference range: 0.5-2.2 mmol/L). Due to ongoing hypotension, the patient was started on a 4 mcg/kg/min dopamine drip in addition to the 1.6 mcg/kg/min norepinephrine drip.

The patient also experienced significant renal impairment with a blood urea nitrogen (BUN) of 27 mg/dL, creatinine of 2.93 mg/dL, and an estimated glomerular filtration rate of 23 mL/min/1.73 m2. In the setting of CCB toxicity, the patient was also started on a calcium chloride infusion, glucagon, and a 0.5 u/kg/hr insulin drip with a dextrose infusion. Intralipid was also administered to act as a lipid sink and prevent further effects of amlodipine, which is highly lipophilic.

**Figure 1:**
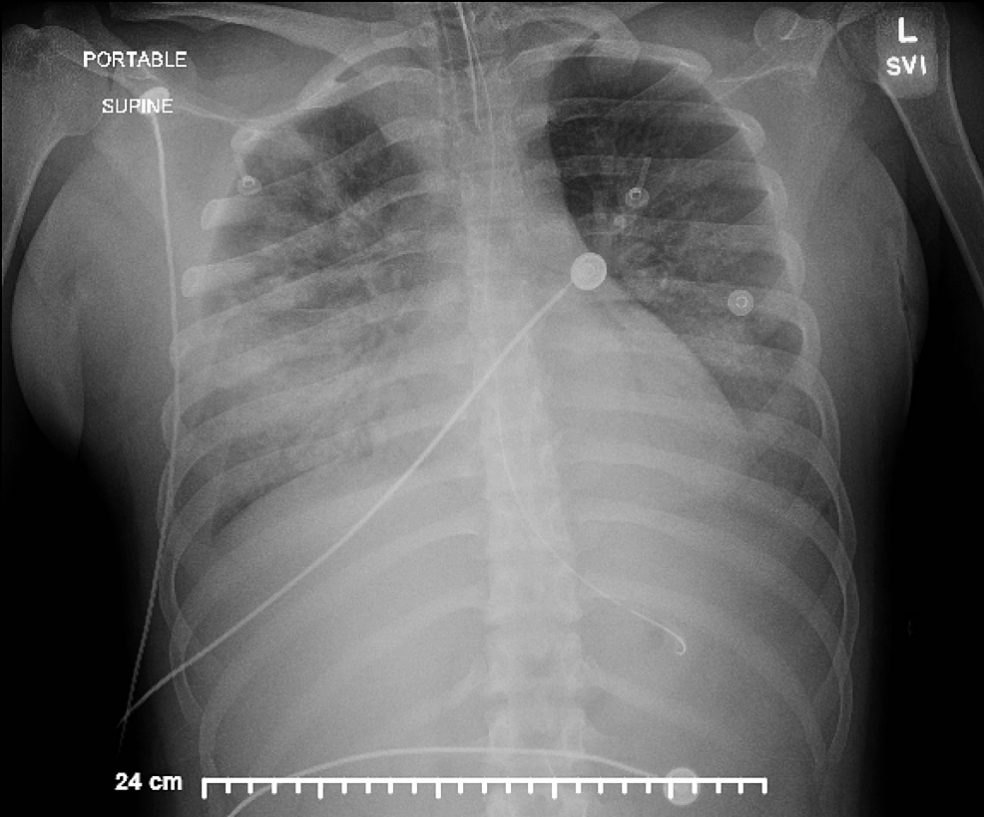
The chest X-ray showed bilateral lung opacities.

Due to her high risk for acute decompensation, the patient was transferred to a higher-level facility in anticipation of needing extracorporeal membrane oxygenation (ECMO) for pulmonary support. Upon arrival, the patient’s blood pressure was 110 mmHg systolic over 38 mmHg diastolic, and her pulse was 111 beats per minute. The patient was prone and oxygenating in the low 90s. A chest X-ray revealed persistent bilateral lung opacities consistent with severe acute respiratory distress syndrome (ARDS), as seen in Figure 1.

Due to persistent hypoxic-hypercarbic respiratory failure, the patient was transitioned from CMV to high-frequency oscillatory ventilation (HFOV). On telemetry, the patient remained in sinus rhythm with the exception of a 10-beat run of ventricular tachycardia and intermittent premature ventricular contractions (PVCs). The ventricular tachycardia was attributed to the patient’s epinephrine spritzer, which was immediately discontinued.

The dopamine drip was discontinued shortly after admission, and the norepinephrine drip was titrated down to 0.8 mcg/kg/min. The patient required aggressive diuresis with furosemide, chlorothiazide, bumetanide, and acetazolamide. Electrolytes were replaced as needed, and the patient was weaned off all diuretics as her creatinine down-trended. The insulin, calcium chloride, and intralipid drips were continued in the management of calcium channel blocker toxicity and gradually discontinued as the patient’s clinical status improved.

**Figure 2:**
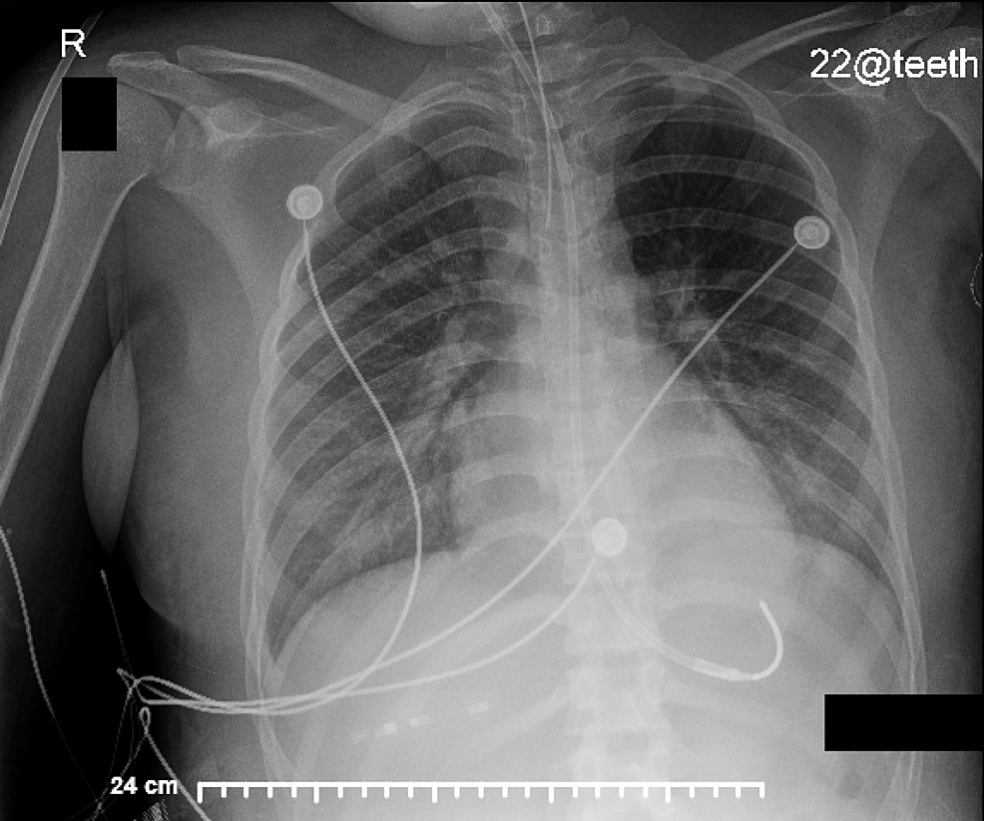
The chest X-ray shows decreased pulmonary edema.

After two days on HFOV, the patient was transitioned back to CMV and was successfully extubated two days later. After extubation, the patient remained on room air for the remainder of her admission without further hypoxemic events. A chest X-ray showed improved aeration of the lungs with decreased edema, as seen in Figure 2.

A renal ultrasound revealed normal kidneys, and the patient’s creatinine appropriately decreased to 0.7 mg/dL. The patient was deemed medically stable for discharge 10 days after her initial presentation to the ED. She was then admitted to an inpatient psychiatric unit.

## Discussion

Acute respiratory distress syndrome is a form of hypoxemic respiratory failure, defined as acute hypoxemia with the presence of bilateral pulmonary infiltrates that cannot be attributed to heart failure or fluid overload. Importantly, our patient received a large volume of fluids upon presentation to the emergency department, which possibly contributed to her acute hypoxic respiratory failure. However, radiographic evidence of acute pulmonary edema in the setting of CCB ingestion and possible aspiration made amlodipine overdose the likely cause of the patient's severe ARDS. It is postulated that immunologic processes involving neutrophils and macrophages mediate inflammatory tissue damage to the bronchial and vascular epithelium, leading to an accumulation of protein-rich fluid in the alveoli. This innate immune response subsequently impairs gas exchange, resulting in hypoxemia [8]. Additionally, the immune cells generate reactive oxygen species, proteases, cytokines, and other inflammatory mediators. These mediators can have a significant impact on type II epithelial cells, the cells that synthesize surfactant in the lungs, which reduces alveolar surface tension [8].

In the setting of CCB toxicity, the pathophysiology of ARDS is not completely understood; however, two possible mechanisms have been proposed. The first of which proposes that calcium channel blockers inhibit surfactant secretion from type II epithelial cells, resulting in alveolar collapse. The second suggests that precapillary vasodilation caused by CCBs results in massive transudation of fluid from pulmonary capillaries into alveoli [5]. Acute respiratory distress syndrome is a relatively rare yet feared complication of CCB overdose, likely due to the drug’s rapid uptake in the body, quick peak plasma concentrations of six-12 hours, long half-life of 30-50 hours, and highly lipophilic properties [9]. There are currently very few case reports detailing the development of ARDS in relation to CCB toxicity in the pediatric population. Jandasek et al. reported a case of amlodipine overdose leading to severe ARDS and shock in a 12-year-old female, which was successfully managed with venovenous ECMO [10].

Amlodipine is a dihydropyridine calcium channel blocker that exerts its vasodilatory effects by blocking L-type calcium channels in vascular smooth muscle cells. The primary concern with CCB overdoses is the severe hypotension and reflex tachycardia that occur as a result of massive vasodilation. It is important to note that this vasodilation was likely amplified by the patient's co-ingestion of tizanidine. Initial management is directed at stabilizing the airway and achieving sufficient circulation. Intravenous crystalloid fluids and vasopressors are commonly used to attain compatible blood pressures and adequate tissue perfusion. Intravenous calcium gluconate or calcium chloride is used in many settings to promote the movement of calcium back into cells via L-type calcium channels. High-dose insulin has been shown to decrease mortality and improve hemodynamics, as CCBs decrease insulin secretion, increase cells’ resistance to insulin, and interfere with glucose metabolism, resulting in lactic acidemia and metabolic acidosis [4]. Lastly, treatment methods such as hemodialysis and other filtration methods are ineffective due to amlodipine’s lipophilicity and significant protein binding capacity [5].

## Conclusions

An intentional, inadvertent, or exploratory overdose of calcium channel blockers can lead to serious, life-threatening complications in the pediatric population, including vasodilatory shock, acute respiratory distress syndrome, and acute kidney injury. Complicating management even further is the lack of treatment modalities available for CCB toxicity. Prompt recognition and judicious management of CCB overdoses can considerably attenuate associated morbidity and mortality.
